# Evaluation of the Different Nutritional and Environmental Parameters on Microbial Pyrene Degradation by Mangrove Culturable Bacteria

**DOI:** 10.3390/ijms24098282

**Published:** 2023-05-05

**Authors:** Manzoor Ahmad, Juan Ling, Jianping Yin, Luxiang Chen, Qingsong Yang, Weiguo Zhou, Yuhang Zhang, Xiaofang Huang, Imran Khan, Junde Dong

**Affiliations:** 1 CAS Key Laboratory of Tropical Marine Bio-Resources and Ecology, Guangdong Provincial Key Laboratory of Applied Marine Biology, South China Sea Institute of Oceanology, Chinese Academy of Sciences, Guangzhou 510301, China; 2 Key Laboratory of Tropical Marine Biotechnology of Hainan Province, Sanya Institute of Ocean Eco-Environmental Engineering, Tropical Marine Biological Research Station in Hainan, Chinese Academy of Sciences, Sanya 572000, China; 3 Guangdong Provincial Observation and Research Station for Coastal Upwelling Ecosystem, South China Sea Institute of Oceanology, Chinese Academy of Sciences, Shantou 515041, China; 4 College of Marine Sciences, South China Agricultural University, Guangzhou 510642, China

**Keywords:** pyrene, nutrient supplements, heavy metals, co-contamination, co-culture, degradation

## Abstract

Mangrove ecosystems play curial roles in providing many ecological services and alleviating global climate change. However, they are in decline globally, mainly threatened by human activities and global warming, and organic pollutants, especially PAHs, are among the crucial reasons. Microbial remediation is a cost-effective and environmentally friendly way of alleviating PAH contamination. Therefore, understanding the effects of environmental and nutritional parameters on the biodegradation of polycyclic aromatic hydrocarbons (PAHs) is significant for the bioremediation of PAH contamination. In the present study, five bacterial strains, designated as Bp1 (Genus *Rhodococcus*), Sp8 (Genus *Nitratireductor*), Sp13 (Genus *Marinobacter*), Sp23 (Genus *Pseudonocardia*), and Sp24 (Genus *Mycolicibacterium*), have been isolated from mangrove sediment and their ring hydroxylating dioxygenase (RHD) genes have been successfully amplified. Afterward, their degradation abilities were comprehensively evaluated under normal cultural (monoculture and co-culture) and different nutritional (tryptone, yeast extract, peptone, glucose, sucrose, and NPK fertilizer) and environmental (cetyl trimethyl ammonium bromide (CTAB), sodium dodecyl sulfate (SDS)) parameters, as well with different co-contaminants (phenanthrene and naphthalene) and heavy metals (Cd^2+^, Cu^2+^, Fe^3+^, Ni^2+^, Mg^2+^, Mn^2+^, and Co^2+^). The results showed that strain Sp24 had the highest pyrene degradation rate (85%) in the monoculture experiment after being cultured for 15 days. Adding nitrogen- and carbon-rich sources, including tryptone, peptone, and yeast extract, generally endorsed pyrene degradation. In contrast, the effects of carbon sources (glucose and sucrose) on pyrene degradation were distinct for different bacterial strains. Furthermore, the addition of NPK fertilizer, SDS, Tween-80, phenanthrene, and naphthalene enhanced the bacterial abilities of pyrene removal significantly (*p* < 0.05). Heavy metals significantly reduced all bacterial isolates’ degradation potentials (*p* < 0.05). The bacterial consortia containing high bio-surfactant-producing strains showed substantially higher pyrene degradation. Moreover, the consortia of three and five bacterial strains showed more degradation efficiency than those of two bacterial strains. These results provide helpful microbial resources for mangrove ecological remediation and insight into optimized culture strategies for the microbial degradation of PAHs.

## 1. Introduction

Mangrove ecosystems constitute an essential and productive ecosystem at the coastal line of the marine environment, providing various ecological and economic services. However, the vast expanse of industrialization and other highly intensive anthropogenic activities have threatened the sustainability and resilience of the mangrove ecosystem, owing to the discharge of ample amounts of pollutants, particularly PAHs [1,2,3]. 

Polycyclic aromatic hydrocarbons (PAHs) are a priority concern pollutant due to their toxic, embryotoxic, and carcinogenic effects, and their ability to bio-accumulate in the biological tissue of plants and animals [4,5,6,7,8]. The United States Environmental Protection Agency (USEPA) has enlisted 16 PAHs as priority pollutants based on their toxic attributes [9]. PAHs are introduced to the environment through diverse sources, such as coal burning, petroleum products, incomplete combustion of organic matter, vehicle emissions, and so on [10]. They could be subdivided into low molecular weight (LMW) and high molecular weight (HMW) PAHs. 

Recently, the investigation of PAHs and the risk associated with the increasing concentration of these toxic compounds is a hot topic due to their detrimental effects on mangrove plants and the surrounding biota [8]. Removing PAHs from the contaminated environment is essential for a healthy and sustainable ecosystem. Yet, the shelf life and the recalcitrancy of PAHs are directly proportional to the molecular size of the compound. At the same time, while the degradation is inversely related to the size of PAHs [11,12], the low degradability of HMW PAHs posed an increased threat to the ecosystems, thus inhibiting the polluted ecosystem’s recovery.

In the environment, PAHs can be removed by volatilization, chemical oxidation, photo-oxidation, bioaccumulation, and by other ways. However, microbial degradation is considered one of the most effective and environmentally friendly methods of PAH removal [13]. Pyrene, a typical four-ring HMW PAHs, is often used to investigate the microbial capabilities of HMW-PAHs degradation. Several bacterial genera have been reported as capable of pyrene degradation. For instance, *Mycobacterium* spp. has been reported as a significant degrader of pyrene in diverse environments [14,15,16,17]. Recently, the deployment of genomic, proteomic, and metabolic approaches has offered intensive insight into pyrene degradation by *Mycobacterium* spp. [18,19]. Similarly, many other pyrene degrading bacterial groups, such as *Pseudomonas* [20], *Bacillus* [21], *Sphingomonas* [22], *Achromobacter* [23], and *Thalassospira* [24], have been reported. Moreover, different techniques, for instance, co-culture [25], co-digestion [24], and the use of bio-surfactant [26], have been adopted to investigate the pattern of pyrene and other forms of PAH degradation. These studies reported that using different culture strategies could affect the biodegradation of PAHs. 

In addition, various environmental factors and nutrient supplements can either enhance or reduce microbial degradation of PAHs. It is crucial to understand the impact of environmental conditions, carbon and nitrogen sources, and contaminants such as heavy metals, as well as the effects of mixed cultures, on the degradation of PAHs. Developing enhanced bio-degradation procedures requires this understanding. Despite the importance of pyrene degradation, limited information is available on the impact of various culture parameters such as nutrients, surfactants, metal, NPK fertilizer, and co-culture. Further research is necessary to better understand the effects of these parameters on pyrene degradation.

Mangrove ecosystems are highly populated with microorganisms capable of degrading polycyclic aromatic hydrocarbons, making them an ideal environment for PAH microbial degradation [12,27]. Therefore, the bacterial strains capable of pyrene degrading were enriched for isolation and pyrene degrading ability measurement in the mangrove ecosystem in this study. Afterwards, strains with high degrading ability were selected for further analysis under varying conditions, including additional carbon/nitrogen sources, surfactants, heavy metals, co-contaminants with other types of PAHs, and co-culture. The study’s results provide valuable insights into the impact of biotic and abiotic factors on the microbial biodegradation of pyrene and are essential for a successful bioremediation plan aimed at restoring and sustaining the mangrove ecosystem. In addition, this study can contribute to the protection of the mangrove ecosystem and promote its restoration.

## 2. Results and Discussion

### 2.1. Identification of Bacterial Strains and Pyrene Degradation

Five bacterial strains (Bp1, Sp8, Sp13, Sp23, and Sp24) were selected for pyrene-degradation experiments based on their relatively fast growth in pyrene-supplemented CFMM medium and successful amplification of the RHD gene. These bacterial strains were preliminarily differentiated by their different colony morphologies on the M8 agar medium (Figure S1). Sequence analyses of the 16S rRNA genes showed that Bp1, Sp8, Sp13, Sp23, and Sp24 were closely related to *Rhodococcus electrodiphilus* (99.13%), *Nitratireductor aquimarinus* (99%), *Marinobacter gudaonensis* (99.23%), *Pseudonocardia carboxydivorans* (99.72%), and *Mycolicibacterium setense* (99%), respectively (Table 1). In the five bacterial strains, the RHD genes were successfully amplified (Figure S2). Among the RHD genes of the five bacterial strains, the sequence results of the three strains (Sp8, Sp13, and Sp24) showed the most relative similarity (≤99%) with a *Nid-A-like* gene of *Mycobacterium* sp.*,* whereas those of Sp23 and Bp1 showed the closest resemblance with the PO2 gene of *Mycobacterium* and the phthalate dioxygenase (*phtAa*) of *Rhodococcus* sp. JDC-11 species, respectively (Table 1).

**Figure 1:**
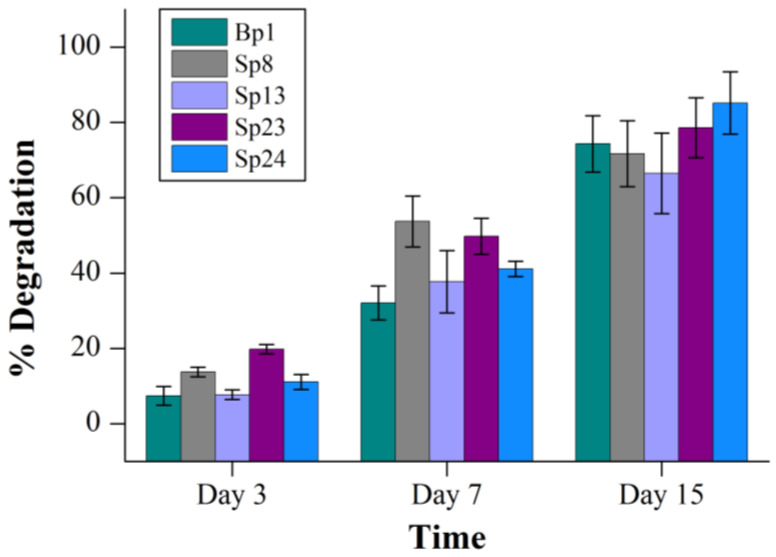
Pyrene degradation by individual bacterial strains. The % degradation of each bacterial strain is the subtracted value of “pyrene lost in experimental group–abiotic pyrene lost in the negative control group” at the corresponding time. The error bar represents the standard deviation.

Among these strains, Sp24 showed the highest degradation of pyrene (85%) after 15 days of incubation, followed by Sp23 (78%) on day 15 (Figure 1). The members of the genus *Mycobacterium* are well-known hydrocarbon degraders and the pyrene degradation by several *Mycobacterium* spp. had been studied extensively. Pyrene could be degraded completely by two *Mycobacterium* strains, PO1 and PO2, after six days [25]. Moreover, the closest resembling strain, *Pseudonocardia carboxydivorans,* with Sp23, was isolated from a soil sample and capable of carbon monoxide oxidation [28]. However, there is no report on PAH degradation by this bacterium; here, we report for the first time the pyrene degradation for this bacterial strain. Similarly, a DNA stable isotope probing investigation by Chen et al. [29] reported that the uncultured *Pseudonorcardia* group was a highly dominant group in a pyrene-treated agricultural soil. Moreover, the pyrene degradation of strains Bp1 and Sp8 were 74% and 71%, respectively (Figure 1), whereas the lowest pyrene degradation (66%) was recorded for strain Sp13 after 15 days of incubation (Figure 1). The members of Genus *Marinobacter* and *Rhodococcus* were important PAH degraders and have previously been reported in many studies [30,31,32,33,34]. Moreover, members of the genus *Nitratireductor* have been isolated from diverse environments, and no reports on PAH degradation by these bacteria are available thus far, although some members of *Nitratireductor*, such as *Nitratireductor lucknowense*, were previously studied from pesticides contaminated sites [35]. The results also demonstrated that pyrene degradation was time-dependent, as low degradation was noticed in the first three days of incubation for each bacterial strain (Figure 1). Similarly, a steady increase in the biomass in terms of OD600 values was also observed with the increasing degradation of pyrene (Figure S3).

### 2.2. Effect of Nutrient Supplements on Pyrene Degradation

**Figure 2:**
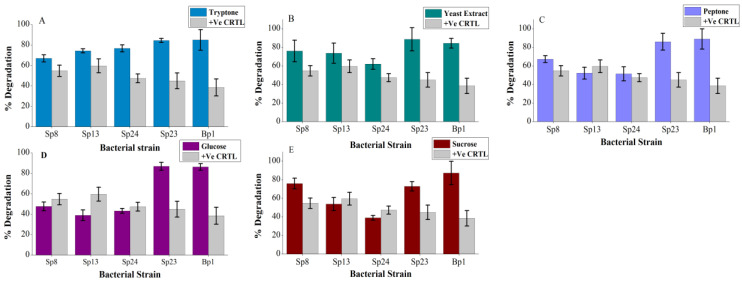
Effect of additional nutrient sources on pyrene degradation: (**A**) tryptone, (**B**) yeast extract, (**C**) peptone, (**D**) glucose, and (**E**) sucrose. The % pyrene degradation represents the subtracted value of “pyrene lost in experimental group–abiotic pyrene lost in the negative control group”. The +Ve CRTL represent pyrene degradation by the corresponding strains without the addition of supplementary carbon and nitrogen source. The error bar represents the standard deviation.

Although pyrene is composed of carbon and hydrogen and can provide carbon and energy for bacteria, the assimilation of pyrene and other HMW PAHs requires a complex enzymatic system. Therefore, HMW PAHs hinder the growth of microorganisms. Additional nutrient supplements such as carbon and nitrogen might be needed for them to promote degradation and rapid microbial growth. The present work showed that additional nutrients (glucose, sucrose, tryptone, peptone, and yeast extract) have a variable impact on the pyrene degradation of different bacterial strains (Figure 2). For instance, the additional nutrient sources significantly enhanced (*p* < 0.05) pyrene degradation of Sp23 and Bp1 bacterial strains (Figure 2) (Table S1). Peptone slightly inhibited pyrene degradation (from 59% to 51%) of Sp13, while a minor increase in pyrene degradation was observed for Sp8 and Sp24 (from 54% to 67% and from 47% to 52%) (Figure 2C). Similarly, in the presence of glucose, the pyrene degradation potential of Sp8, Sp13, and Sp24 was reduced by up to 7%, 21%, and 4%, respectively (Figure 2D). Moreover, sucrose also showed an inhibitory effect on the pyrene degradation of Sp13 and Sp24 bacterial strains (Figure 2E). The high growth rate in terms of OD_600_ was recorded for Bp1 and Sp23 bacterial strains in the presence of all nutrient sources (Figure S4). In two independent studies, the negative effect of glucose on phenanthrene and pyrene degradation was investigated previously by Wong et al. [36] and Patel, Mahala, Jain, and Madamwar [26]. Our results suggested that the effect of additional carbon sources was not uniform, and the response of different strains was different. Similarly, the relatively high pyrene degradation by Bp1 and Sp23 (Figure 2) in the presence of an additional nitrogen source might be due to their robust growth. Several former studies reported the nitrogen rich supplement’s synergistic effect on the degradation of PAHs [37,38,39]. Patel, Mahala, Jain, and Madamwar [26] reported that peptone was the most effective enhancer of PAH degradation by DAK11 bacterial consortia. It is worth mentioning that peptone slightly reduced the pyrene degradation efficiency of strain Sp13, which was consistent with the previous results of Zhou et al. [24].

### 2.3. Effect of Surfactant on Pyrene Degradation

**Figure 3:**
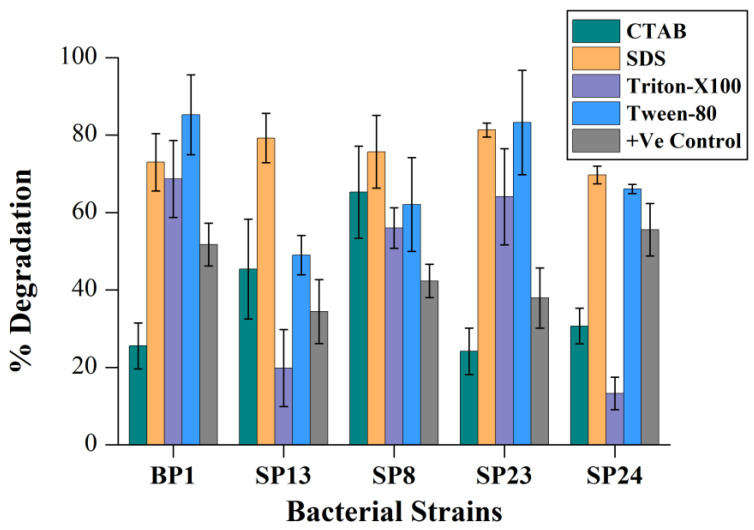
Effect of surfactant on pyrene degradation. The % pyrene degradation represents the subtracted value of “pyrene lost in experimental group–abiotic pyrene lost in the negative control group”. The +Ve CRTL represents pyrene degradation by the corresponding bacterial strains without the addition of surfactants. The error bar represents the standard deviation.

The bioavailability of PAHs is the rate-limiting factor of their degradation because of hydrophobicity. Generally, some microorganisms produce surface-active agents (bio-surfactants) which can solubilize these compounds and make them available for assimilation. Using external surfactants (sodium dodecyl sulfate (SDS), cetyl trimethyl ammonium bromide (CTAB), Tween 80, and Triton X100) could increase the solubility and bioavailability of PAHs; hence, they can increase the biodegradation. Our results demonstrated that different surfactants had a discrete effect on pyrene degradation and were highly strain dependent. The addition of CTAB resulted in a 16–25% decrease in pyrene degradation by Bp1, Sp23, and Sp24 strains compared to their positive control (Figure 3), while an increase (11–20%) in pyrene degradation by Sp8 and Sp13 was also observed (Figure 3). These results suggest that CTAB might inhibit the growth of some bacterial strains. On the other side, the pyrene degradation of all strains increased significantly (*p* < 0.05) in the presence of SDS (Table S1) (Figure 3). These results were in line with those findings of Patel et al. [26]. Moreover, the addition of Tween-80 significantly enhanced the pyrene degradation abilities (*p* < 0.05). However, the effect of Tritoon-X100 was strain dependent. It increased the pyrene degradation efficacy of strains Bp1, Sp8, and Sp23 while decreasing the degradation abilities of strains Sp13 and Sp24 (Figure 3). The different growth patterns of bacteria in the presence of various surfactants also suggested that different surfactants had distinct effects on pyrene degradation (Figure S5). The enhanced consumption of pyrene by strains Bp1, Sp8, and Sp23 in the presence of nonionic surfactants Triton X100 and Tween-80 indicated that these bacterial strains could assimilate these surfactants along with pyrene (Figure 3). According to previous studies, the degradation effect of nonionic surfactants on PAHs was better than that of anionic and cationic surfactants. For instance, Makkar and Rockne [40] reported that the degradation of naphthalene, pyrene, and phenanthrene increased after adding Triton X100. Similarly, Tween-80 addition also increased the degradation of phenanthrene by *Pseudoxanthomonas* sp. DMVP2 [38].

### 2.4. Effect of Heavy Metals on Pyrene Degradation

**Figure 4:**
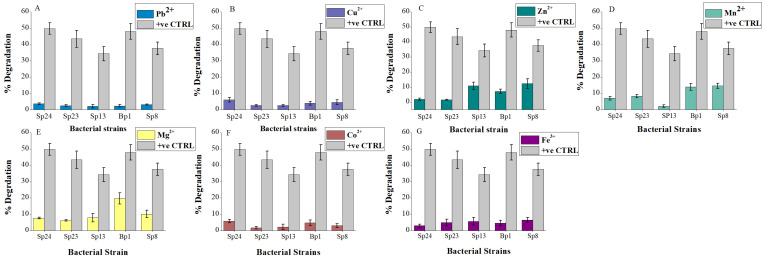
Effect of heavy metal on pyrene degradation: (**A**) Pb^2+^, (**B**) Cu^2+^, (**C**) Zn^2+^, (**D**) Mn^2+^, (**E**) Mg^2+^, (**F**) Co^2+^, and (**G**) Fe^3+^. The % pyrene degradation represents the subtracted value of “pyrene lost in experimental group–abiotic pyrene lost in the negative control group”. The +Ve CRTL represents pyrene degradation by the corresponding bacterial strains without the addition of heavy metals. The error bar represents the standard deviation.

Heavy metal addition significantly reduced (*p* < 0.05) pyrene degradation for all bacterial strains (Figure 4) (Table S1). In general, adding different heavy metals reduced the pyrene degradation capacities by up to 22% to 45% of the studied bacterial strains (Figure 4). The isolates, Sp8, Bp1, and Sp13, showed 8%, 12%, and 14% pyrene degradation after nine days of incubation in the presence of Zn^2+^ (Figure 4C). Similarly, in the presence of Mn^2+^ highest degradation, 14% and 16% were recorded for Bp1 and Sp8, respectively (Figure 4D). Moreover, Bp1 showed 20% degradation of pyrene in the presence of Mg^2+^ (Figure 4E). These inhibitions may be due to the toxic effect of heavy metals on bacterial strains. Compared to the mild pyrene degradation of their positive control, strains Bp1 and Sp8, with Zn^2+^, Mn^2+^, and Mg^2+^, reflected their resistance to these metals (Figure 4C–E). Similar results of reduced PAH degradation in the presence of heavy metals were reported by Patel et al. [26]. Similarly, Deary et al. [41] reported 20.7%, 27.7%, and 37.6% PAH removal inhibition in the presence of Pb, Cd, and Hg, respectively.

### 2.5. Effect of Co-Contaminants

**Figure 5:**
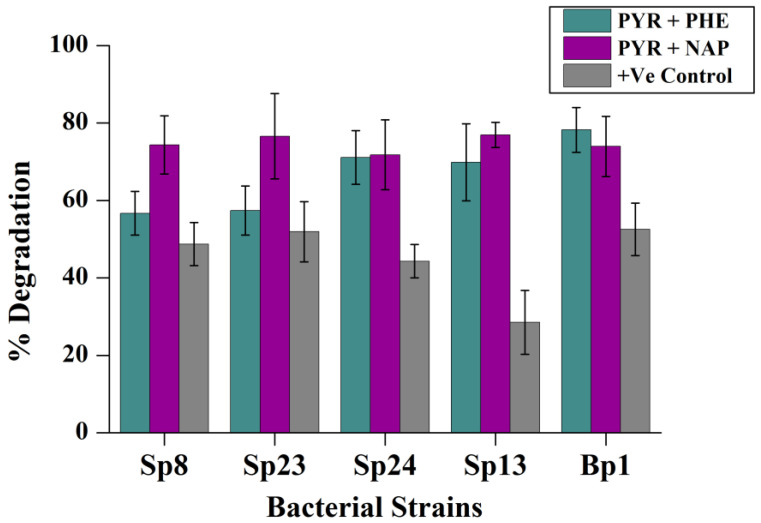
Pyrene degradation in the presence of low molecular weight phenanthrene and naphthalene provided with a concentration of 50 mg/L. The % pyrene degradation represents the subtracted value of “pyrene lost in experimental group–abiotic pyrene lost in the negative control group”. The +Ve CRTL represents pyrene degradation by the corresponding bacterial strains without the addition of phenanthrene and naphthalene. The error bar represents the standard deviations.

The presence of additional different PAHs exhibited a positive effect on pyrene degradation. The addition of 50 mg/L phenanthrene significantly increased the pyrene degradation by Bp1, Sp13, and Sp24 (*p* < 0.05) (Table S1). A marked increase was observed up to 60% (28% to 89%) in the presence of phenanthrene by Sp13 compared with its positive control, followed by strain Sp8 and Sp23 (Figure 5). Similarly, the addition of 50 mg/L naphthalene showed a synergistic effect on pyrene degradation of all strains (Figure 5). Similar results of enhanced degradation of HMW PAHs by the addition of readily available low molecular PAHs have been observed previously [42]. These results suggested that low molecular weight PAHs could stimulate the enzymatic cascade of PAH degradation and increase the number of bacterial cells, thus enhancing the degradation rate of HMW PAHs. As mentioned by Durant and Bouwer [43], co-metabolism involves the conversion of non-growth substrates by growing active cells in the presence of growth substrates and resting cells in the presence of an energy substrate. The relatively high biomass in terms of OD_600_ values of the five bacterial strains in the phenanthrene and naphthalene-amended group also indicates that low molecular PAHs synergistically affect the degradation of HMW PAHs (Figure S6). 

### 2.6. Effect of NPK Fertilizer on Pyrene Degradation

**Figure 6:**
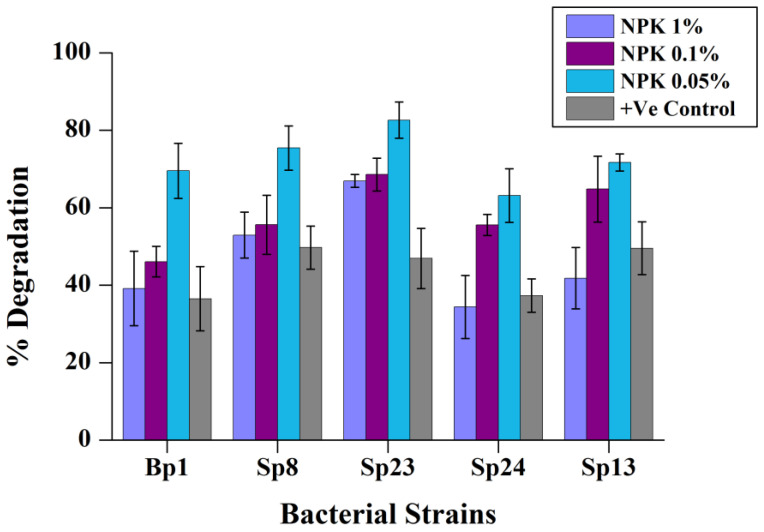
Effect of different NPK fertilizer concentrations on pyrene degradation. The % pyrene degradation represents the subtracted value of “pyrene lost in experimental group–abiotic pyrene lost in the negative control group.” The +Ve CRTL represents pyrene degradation by the corresponding bacterial strains without the addition of NPK fertilizer. The error bar represents the standard deviation.

**Figure 7:**
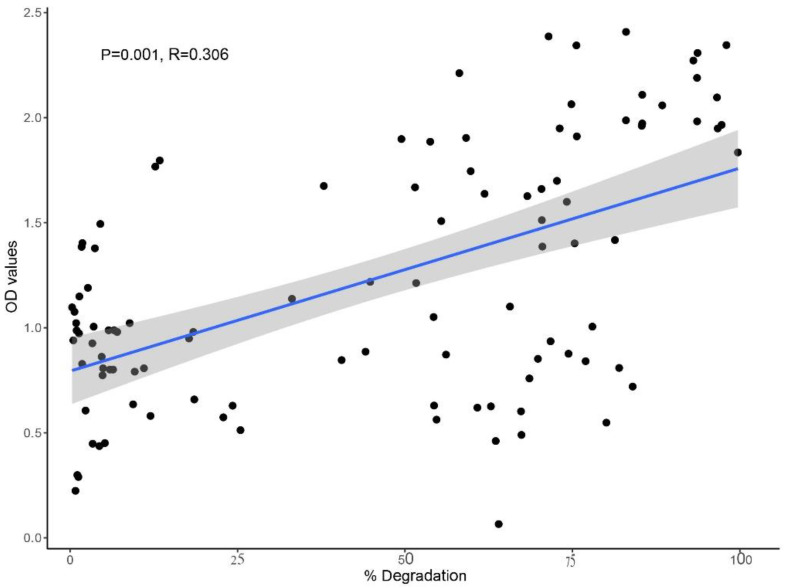
Correlation analysis between bacterial cell growth in terms of OD values and % degradation of pyrene.

Previous studies showed that the addition of NPK fertilizer increased the PAHs degradation [44,45] and might be used for in situ bioremediation at the contaminated site. However, the removal of PAHs in the soil is affected by the extent and the combination of fertilizer Fu et al. [46]. Therefore, it is essential to investigate the optimal NPK fertilizer concentration for maximum PAHs degradation. Our results showed that the addition of NPK fertilizer had a positive effect on pyrene degradation for most of the five bacterial strains. In addition, the pyrene degradation decreased once the NPK amount exceeded the optimum concentration. Significantly enhanced (*p* < 0.05) degradation of pyrene was recorded at 0.05% concentration (N:P:K 15:15:15) (Table S1) (Figure 6). Additionally, strain Sp23 showed the highest degradation at each concentration of NPK (Figure 6). Our results of the highest degradation at a concentration of 0.05% NPK are consistent with the former reports [26]. All strains’ relatively high growth rate at 0.05% NPK concentration and significant correlation between pyrene removal rate and OD values could provide insight into the NPK synergistic effect on pyrene degradation (Figure 7 and Figure S7).

### 2.7. Effect of Co-Culture on Pyrene Degradation

**Figure 8:**
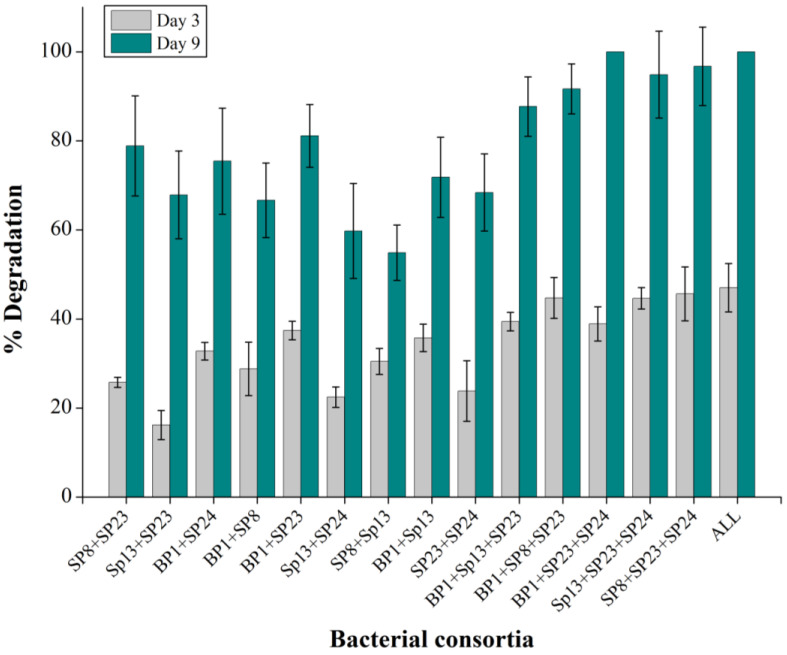
Pyrene degradation by the consortia of two, three, and five bacterial strains of the study isolates. Full (100%) degradation was considered for the treatment in which no pyrene was detected in the HPLC analysis. The % pyrene degradation represents the subtracted value of “pyrene lost in experimental group–abiotic pyrene lost in the negative control group”. The error bar represents the standard deviation.

The results of the drop collapse method demonstrated the highest bio-surfactant production for Bp1 and Sp23 (+++), followed by Sp13 and Sp8 (++), and the lowest one for Sp24 (+) (Table 1). A positive trend was observed between the consortium with a high potential for bio-surfactant production and pyrene degradation. Many studies reported that the co-culture strategy of PAHs degradation was the most effective way [25,26]. Bp1 + Sp23 consortia exhibited enhanced pyrene degradation, and the degradation rates on the third and ninth day were 37% and 81%, respectively (Figure 8). Similarly, combining with any one of the Bp1 or Sp23 consortiums showed enhanced activities for pyrene degradation. The relatively high degradation of pyrene by the bi-culture consortia containing Bp1 and Sp23 indicated that high-producing bio-surfactant strains could increase pyrene degradation. This may be because that bio-surfactant increased the solubility of pyrene and made it available for the low/non-producing bio-surfactant bacterial strains. It has been reported that the inclusion of bio-surfactant-producing *Bacillus* sp. WF1 increased the pyrene degradation efficiency of *Mycobacterium* strains PO1 and PO2 [25]. The consortia’s Bp1 + Sp24 and Sp23 + Sp8 showed 74% and 78% pyrene degradation after nine days of incubation (Figure 8). On the other side, 100% pyrene degradation was achieved on the ninth day of incubation when a consortium of Bp1 + Sp23 + Sp24 was employed, which were previously confirmed and designated as high bio-surfactant producing strains. In the culture of these consortia, no peaks related to pyrene were detected in HPLC, suggesting that the pyrene has been completely degraded. The comparatively high pyrene degradation by the consortia of three and five bacterial strains compared to bi-culture consortia indicates that PAHs-degrading bacteria have established a synergistic association (Figure 8). Ghazali et al. [47] mentioned that the degradation of pollutants by using the microbial consortia was not merely the sum of individual strain’s degradation efficiency; the increased degradation of contaminants could be attributed to the synergistic association of consortia [48]. Similarly, Arulazhagan, Vasudevan, and Yeom [39] reported that the consortia containing *Ochrobactrum sp*, *Enterobacter cloacae*, and *Stenotrophomonas maltophilia* degraded above 95% naphthalene, phenanthrene, and pyrene after four days of incubation. High bacterial growth in term of OD values has been observed in the group with high pyrene degradation potential (Figure S8). Moreover, correlation analysis also showed a significant correlation between pyrene degradation and bacterial growth rate (Figure 7).

## 3. Material and Methods

### 3.1. Chemical and Media

Pyrene, acetone, ethyl acetate, and methanol used in this study were of high purity grade, procured from Aladine industrial corporation, Shanghai, China. The CFMM (carbon-free minimal medium) was prepared by adding Na_2_HPO_4_ 2.2 g, NH_4_NO_3_ 1 g, K_2_HPO_4_ 1 g, MgSO_4_ 0.2 g, FeCl_3_ 0.05 g, and CaCL_2_ 0.02 g in 1 L of distilled water. The CFMM was supplemented with a filter-sterilized trace element solution containing 3 mg MnSO_4_, 3 mg ZnSO_4_, 1 mg CoSO_4_, and 1 mg (NH_4_)_6_Mo_7_O_2_ per liter of distilled water. The M8 agar medium used for the isolation of bacterial strains was prepared by adding CH_3_COONa 2.0 g, NH_4_NO_3_ 1 g, Tryptone 0.5 g, Yeast extract 0.5 g, glucose 0.5 g, sucrose 0.5 g, potato extract 0.5 g, KH_2_PO_4_ 0.5 g, NH_4_Cl 0.2 g, sodium citrate 0.05 g, malic acid 0.05 g, and 15 g agar [30]. 

### 3.2. Sample Collection, Enrichment and Isolation of Pyrene Degrading Bacteria

Surface sediment samples (0–10 cm) were collected from a mangrove forest in triplicate at Hongsha river (18.2629° N, 109.5772° E), Sanya, Hainan Island, China. Table S2 shows the physiochemical characteristics of the collected samples. The sediment was transferred to the laboratory on the ice as soon as possible and processed as soon as possible. A composite sample was prepared by thoroughly mixing part of the three samples. Initially, the sediment samples were enriched by providing pyrene as the sole carbon source. The enrichment was performed in a 250 mL flask containing 100 mL carbon free minimal medium (CFMM) supplemented with pyrene (100 mg/L) and a 5 g wet sediment sample. Before the addition of the sediment sample, pyrene dissolved in acetone was evaporated by keeping the flasks overnight in a laminar flow hood. The flasks were covered with aluminum foil and incubated in a shaker at 30 °C and 180 rpm for 20 days. After the first incubation, 10 mL supernatant was transferred to fresh CFMM containing 100 mg/L pyrene and incubated as before. After five successive enrichments, 1 mL of the final enrichment culture was serially diluted and spread on M8 solid agar plates. The plates were incubated for two weeks at 30 °C. Morphologically distinct colonies were picked up and purified. Each bacterial strain was identified based on 16S rDNA by the universal primer set 27F and 1492R. The bacterial strains were identified against the EZBioCloud database (https://www.ezbiocloud.net/) (accessed on 25 December 2021) for similarity search. 

The bacterial strains were further screened for the presence of the ring hydroxylating dioxygenase (RHD) gene, involved in the initial cleavage of PAHs ring. The study isolates used a nested PCR primer set [49] for RHD gene amplification [50]. The primers sequence of the first PCR round was NID-for (TCCRMTGCCCDTACCACGG)/NID-rev1 (GAASGAYARRTTSGGGAACA), and NID-rev2 (GCGSCKRKCTTCCAGTTCG) was applied for nested PCR round. The first-round PCR reaction was performed in a 25 µL reaction mixture containing 12.5 µL Ex Taq (Premix), 1 µL of each primer, 0.5 µL BSA (Bovine serum albumin), 1 µL template DNA, and 10.5 µL PCR water. The product of the first PCR reaction was used as a template for the second nested round of PCR. The PCR conditions for the first round were 94 °C for 3 min, 40 cycles of 94 °C for 45 s, 55 °C for 45 s, and 72 °C for 45 s and a final extension at 72 °C for 7 min. The PCR conditions were the same for the nested round; only the PCR cycles were reduced to 30. The product of the nested round of PCR was subjected to gel electrophoresis, and the amplicons of around 300 bp were sequenced. The sequences were blast searched in the NCBI database for similarity search.

### 3.3. Pyrene Degradation Experiment

Five bacterial strains, designated as Sp8, Sp13, Sp23, Sp24, and Bp1, were used for pyrene degradation experiments. Each bacterial isolate was grown in M8 broth to the late log phase for pyrene degradation. Cells were harvested by centrifugation and washed twice with 0.85% normal saline. Finally, the cell pellets were suspended in CFMM to adjust the OD to 0.2 and used as inoculum for pyrene degradation. 

The experiment was performed in a 50 mL flask containing 18 mL CFMM provided with 100 mg/L pyrene as a carbon source and 2 mL of individual bacterial strain cell suspension. The flasks were prepared in a replicate of nine for each bacterial strain, covered with aluminum foil and incubated in a rotary shaker (180 rpm) at 30 °C for 15 days. Simultaneously, controls were placed containing CFMM and the same concentration of pyrene, aiming to depict the abiotic loss of pyrene during the experimental process. The pyrene degradation was measured after 3, 7, and 15 days of incubation by taking out triplicate flasks.

The residual pyrene in each flask was extracted with two volumes of ethyl acetate twice, dehydrated with anhydrous sodium sulfate, dried in a rotary evaporator, and finally dissolved in methanol. High-Performance Liquid Chromatography (HPLC) analyzed the final methanol extract using an intertStill, ODs 4.6 × 150 mm column. The mobile phase used was 80% methanol with a flow rate of 1 mL minute at 275 nm. A standard curve of pyrene with R^2^ of 0.99 was generated, and the concentration of pyrene at different time points was calculated. The degradation of pyrene for each bacterial isolate was calculated according to the following formula described earlier [25]:

$$\%Pyrene\;degradation=\frac{Cpyr-Epyr}{Ipyr}\times 100$$

where *Cpyr* is the pyrene concentration in the control group, *Epyr* represents pyrene concentration in the experimental group, and *Ipyr* is the initial concentration of pyrene.

### 3.4. Effect of Nutrient Supplements on Pyrene Degradation

The effect of additional carbon- and nitrogen-rich sources on pyrene degradation was studied for each bacterial strain. A total of 100 mg/L CFMM containing pyrene was supplemented with 0.1% either with carbon- (glucose or sucrose) or nitrogen-rich sources (tryptone, peptone, or yeast extract). Similarly, positive control (composed of CFMM + Pyrene + one of the studied strains) and negative control (CFMM + Pyrene) were placed in parallel. The flasks were prepared in triplicate, covered with aluminum foil, and incubated in a rotary shaker (180 rpm) at 30 °C for nine days. The residual pyrene and the pyrene degradation were calculated according to the above procedure. The bacterial growth was monitored at OD_600_.

### 3.5. Effect of Surfactant on Pyrene Degradation

Different surfactants were used in the present study to investigate the effect of surfactant on pyrene degradation. Surfactants such as sodium dodecyl sulfate (SDS), triton x100, tween-80, and cetyl trimethyl ammonium bromide (CTAB) were added to CFMM with a concentration of 0.1%. The positive control was prepared by the same procedure as mentioned above. The negative control contains CFMM, 100 mg/L pyrene, and one of the above-mentioned surfactants. All the experimental and control groups were kept under the same conditions during investigation. The above-mentioned procedure has been employed to measure the pyrene degradation after nine days of incubation. The bacterial growth was monitored at OD_600_.

### 3.6. Effect of Heavy Metals on Pyrene Degradation

The effect of different heavy metals on pyrene degradation by bacterial isolates was investigated. Heavy metals such as Cd^2+^, Cu^2+^, Fe^3+^, Ni^2+^, Mg^2+^, Mn^2+^, and Co^2+^ were added to CFMM at a concentration of 1mM in triplicate. The positive and negative controls were prepared following the same procedure, except the negative controls do not have any of the strain responsible for biodegradation. All the established groups were treated simultaneously using the same experimental conditions. The pyrene degradation was measured after nine days of incubation by using the same procedure.

### 3.7. Effect of Co-Contaminants on Pyrene Degradation

The effect of other polycyclic aromatic hydrocarbons on pyrene degradation was investigated in the CFMM containing 100 mg/L pyrene supplemented with 50 mg of either phenanthrene or naphthalene. Each experimental group was prepared in triplicate for individual strains. Positive control contains 18 mL CFMM, 2 mL cell suspension of the individual strain, and 100 mg/L pyrene, while the experimental group contains 20 mL CFMM, 100 mg/L pyrene, and 50 mg/L of either phenanthrene or naphthalene. All the experimental groups, including the control, were incubated under the same physiological conditions. The pyrene degradation was measured after nine days of incubation accordingly. The bacterial growth was monitored at OD_600_.

### 3.8. Effect of NPK Fertilizer on Pyrene Degradation

To investigate the effect of NPK fertilizer on pyrene degradation by five bacterial strains, the NPK fertilizer (composition 15:15:15) was purchased from Lvfeng Fertilizer Co. Ltd., Zibo, China. The CFMM was supplemented with different concentrations (0.1%, 0.5%, and 1%) of NPK fertilizer. The flasks were prepared in triplicate for each concentration. The positive control was run in parallel using the same protocol while for negative control CFMM was supplemented with 100 mg/L pyrene and NPK. All the established groups were processed under the same condition. The pyrene degradation was measured after 9 days of incubations according to the method described earlier, monitoring the bacterial growth at OD_600_.

### 3.9. Effect of Co-Culture on Pyrene Degradation

The effect of co-culture (consortia) with a different combination of tested isolates on pyrene degradation was investigated. The combination of two, three, and five bacterial strains were studied for pyrene degradation. The strain’s combination was based its their capacity for bio-surfactant production. The bio-surfactant production abilities were qualitatively measured by the drop collapse method as previously described by Wan Nawawi et al. [50]. Briefly, each bacterial isolate was grown for three days in CFMM supplemented with 1% soya been oil. After incubation, the culture was centrifuged at 5480× *g* for 15 min. The residual soya bean oil was extracted with an equal volume of hexane, and the two phases were separated by using a separatory funnel. The supernatant was tested for bio-surfactant by drop collapse method. For this, 100 µL supernatant sample was added on a surface of parafilm and observed after one minute. At the same time, 100 µL of CFMM was also added for comparative analysis. The drops beaded up, collapsed, or slightly spread out depending on the extent of the bio-surfactant in the sample. The culture which produces a large diameter of flat droplets was scored as a high producer and designated as ‘**+++**’. Similarly, the supernatant with intact small round droplets was scored as ‘**+**’ and the supernatant with intermediate diameter droplets was scored as ‘**++**’. 

In the bi-culture bacterial consortia, high bio-surfactant producing strains ‘**+++**’ were grouped with ‘**+**’ and ‘**++**’. Similarly, the combination of ‘**+++**’ bacterial strains and ‘**++**’ and ‘**+**’ were also made. Moreover, in the case of a three-bacterial-strain consortia, two ‘**+++**’ were grouped either with ‘**+**’ or ‘**++**’, or two ‘**+**’ or ‘**++**’ were grouped with one ‘**+++**’ bacterial strain. The pyrene degradation experiments were performed in 50 mL flasks containing 18 mL CFMM and 2 mL cell suspension of consortia (equal volume of each isolate’s cell suspension with OD 0.2 was mixed) provided with 100 mg/L of pyrene. The flasks were prepared in a replicate of six for each consortium and incubated for nine days in a rotary shaker. The bacterial growth was monitored at OD_600._ The pyrene degradation was measured after 3 and 9 days of incubation by taking triplicate flasks. 

### 3.10. Statistical Analysis

All the graphs were made using OriginPro 8.5 software (OriginLab Corp., Northampton, MA, USA). A correlational analysis between OD values and % degradation was carried out. The Monte Carlo test was applied on the date set to depict the significant effects of different nutritional and culture conditions on pyrene degradation. These analyses were performed using R (version 4.1.2) (R Core Team, 2020, Vienna, Austria). 

## 4. Conclusions

Five high pyrene-degrading bacteria, Bp1 (Genus *Rhodococcus*), Sp8 (Genus *Nitratireductor*), Sp13 (Genus *Marinobacter*), Sp23 (Genus *Pseudonocardia*), and Sp24 (Genus *Mycolicibacterium*), have been isolated from a mangrove ecosystem and characterized, both at a molecular (ring hydroxylating dioxygenase (RHD) gene) and a physiological level (PAHs degradation), under different nutritional conditions, co-contaminants, and co-culture conditions, respectively. Our investigation revealed that pyrene degradation could be affected (positively or negatively) by nutritional and environmental factors. Additional nutritional carbon (glucose and sucrose) and nitrogen (tryptone, yeast extract, peptone) sources could synergistically affect PAH degradation; however, the effect varied for different bacterial strains. Moreover, it could be inferred that nonionic surfactants produced a more positive impact on pyrene degradation than ionic surfactants. The addition of NPK fertilizer increased pyrene degradation. Similarly, low molecular weight PAHs (phenanthrene and naphthalene) boosted the degradation of HMW PAHs. We concluded from the co-culture experiment that bio-surfactant-producing bacteria could enhance the assimilation of pyrene and other PAHs. Moreover, the significant positive correlation between pyrene degradation and bacterial growth indicated that pyrene could be used as a source of energy and biomass by microorganisms (*p* < 0.05). Further intensive investigations are needed to illustrate the molecular mechanism of microbial community interaction during pyrene degradation and to provide a theoretical and technical foundation for mangrove ecosystem microbial ecological restoration. 

## Figures and Tables

**Table 1:** 16S rDNA and RHD gene taxonomic affiliation of the study bacterial isolates and their bio-surfactant production level.

Strains	Identical Strains	% Similarity of 16S rRNA	Identical RHD Gene	% Similarity RHD Gene	Bio-Surfactant Production
Sp8	*Nitratireductor aquimarinus*	99.00%	*Mycobacterium* sp. py143 *Nid*A	99%	++
Sp13	*Marinobacter gudaonensis*	99.23%	*Mycobacterium* sp. py143 *Nid*A	99%	++
Sp23	*Pseudonocardia carboxydivorans*	99.72%	*Mycobacterium* sp. PO2 genes	96%	+++
Sp24	*Mycolicibacterium setense*	99.00%	*Mycobacterium* sp. py143 *Nid*A	99%	+
Bp1	*Rhodococcus electrodiphilus*	99.13%	*Rhodococcus* sp. JDC-11 Phthalate dioxygenase large subunit (*pht*Aa) gene	99%	+++

## Data Availability

The data supporting these findings can be found in the Supporting Materials.

## Supplementary Materials

The supporting information can be downloaded at https://www.mdpi.com/article/10.3390/ijms24098282/s1.
