# Supplementary material for: Evaluation of the Different Nutritional and Environmental Parameters on Microbial Pyrene Degradation by Mangrove Culturable Bacteria
Source: Int J Mol Sci. 2023 May 5;24(9):8282. doi: 10.3390/ijms24098282 (PMC10179275; doi:10.3390/ijms24098282)
Supplement: Supplementary file 1 [file ijms-24-08282-s001.zip › ijms-2347668-supplementary.pdf]

Evaluation of the Different Nutritional and Environmental Parameters on Microbial Pyrene Degradation by Mangrove Culturable Bacteria

Manzoor Ahmad [1], Juan Ling [1,2,3,*], Jianping Yin [1], Luxiang Chen [4], Qingsong Yang [1,2,3], Weiguo Zhou [1,2], Yuhang Zhang [1,2], Xiaofang Huang [1,2], Imran Khan [1] and Junde Dong [1,2,3,*]

[1] CAS Key Laboratory of Tropical Marine Bio-Resources and Ecology, Guangdong Provincial Key Laboratory of Applied Marine Biology, South China Sea Institute of Oceanology, Chinese Academy of Sciences, Guangzhou 510301, China

[2] Key Laboratory of Tropical Marine Biotechnology of Hainan Province, Sanya Institute of Ocean Eco-Environmental Engineering, Tropical Marine Biological Research Station in Hainan, Chinese Academy of Sciences, Sanya 572000, China

[3] Guangdong Provincial Observation and Research Station for Coastal Upwelling Ecosystem, South China Sea Institute of Oceanology, Chinese Academy of Sciences, Shantou 515041, China

[4] College of Marine Sciences, South China Agricultural University, Guangzhou 510642, China

* Correspondence: lingjuan@scsio.ac.cn (J.L.); dongjd@scsio.ac.cn (J.D.)

Supplementary materials

Table. S1. Monte Carlo test illustrates the significant effect of different treatments on pyrene degradation by five bacterial strains. The significant values with a symbol (↑) and (↓) illustrate the significant increase and decrease of pyrene degradation by the corresponding treatments ($P < 0.05$). All the studied metals significantly inhabited pyrene degradation hence the values shown in the table represents all the studied metals.

**Bacterial Strains**	**Addition nutrient**					**Surfactants**				**NPK**			**Co-Contaminants**		**Heavy Metals**
	**TRP**	**Y. E**	**PEP**	**GLU**	**SUC**	**CTAB**	**SDS**	**T- X100**	**T-80**	**1%**	**0.1%**	**0.05%**	**PHE**	**NAP**	
**Sp8**	0.008 ↑	0.001 ↑	0.005↑	0.142	0.004 ↑	0.005 ↑	0.002 ↑	0.007 ↑	0.023 ↑	0.065 ↑	0.051 ↑	0.004↑	0.022 ↓	0.034 ↑	0.001 ↓
**Sp13**	0.023 ↑	0.021 ↑	0.123	0.077	0.941	0.019 ↑	0.004 ↑	0.003 ↓	0.035 ↑	0.077 ↑	0.025 ↑	0.001↑	0.001 ↑	0.004 ↑	0.001 ↓
**Sp23**	0.001 ↑	0.002 ↑	0.002 ↑	0.002↑	0.078	0.003 ↓	0.001 ↑	0.009 ↑	0.001 ↑	0.002 ↑	0.001 ↑	0.001↑	0.001 ↓	0.007 ↑	0.001 ↓
**Sp24**	0.004 ↑	0.021 ↑	0.451	0.061	0.001↑	0.001 ↓	0.014 ↑	0.002 ↓	0.008↑	0.057 ↑	0.012 ↑	0.005↑	0.001 ↑	0.023 ↑	0.001 ↓
**Bp1**	0.001 ↑	0.001↑	0.001 ↑	0.001↑	0.001↑	0.008 ↓	0.001 ↑	0.001 ↑	0.006 ↑	0.006 ↑	0.033 ↑	0.004↑	0.001 ↑	0.009 ↑	0.001 ↓

TRP: Tyotone, Y.E: Yeast Extract, PEP: Peptone, GLU: Glucose, CTAB: Cetyl trimethyl ammonium bromide, SDS: Sodium dodecyl sulfate, PHE: Phenanthrene, NAP: Naphthalene; Metal: Cd^{2+}, Cu^{2+}, Fe^{3+}, Ni^{2+}, Mg^{2+}, Mn^{2+} and Co^{2+}

Table. S2. Illustrating the physiochemical characteristic of the collected mangrove sediment samples

**Sample name**	**pH**	**Total organic carbon (g/kg)**	**Total nitrogen (g/kg)**	**Total phosphorus (g/kg)**	**Total water-soluble salts (g/kg)**
Sample_1	6.93	29.5	1.89	0.17	2.08
Sample_2	7.05	21.6	1.35	0.23	3.74
Sample_3	7.08	43.2	2.68	0.60	7.88

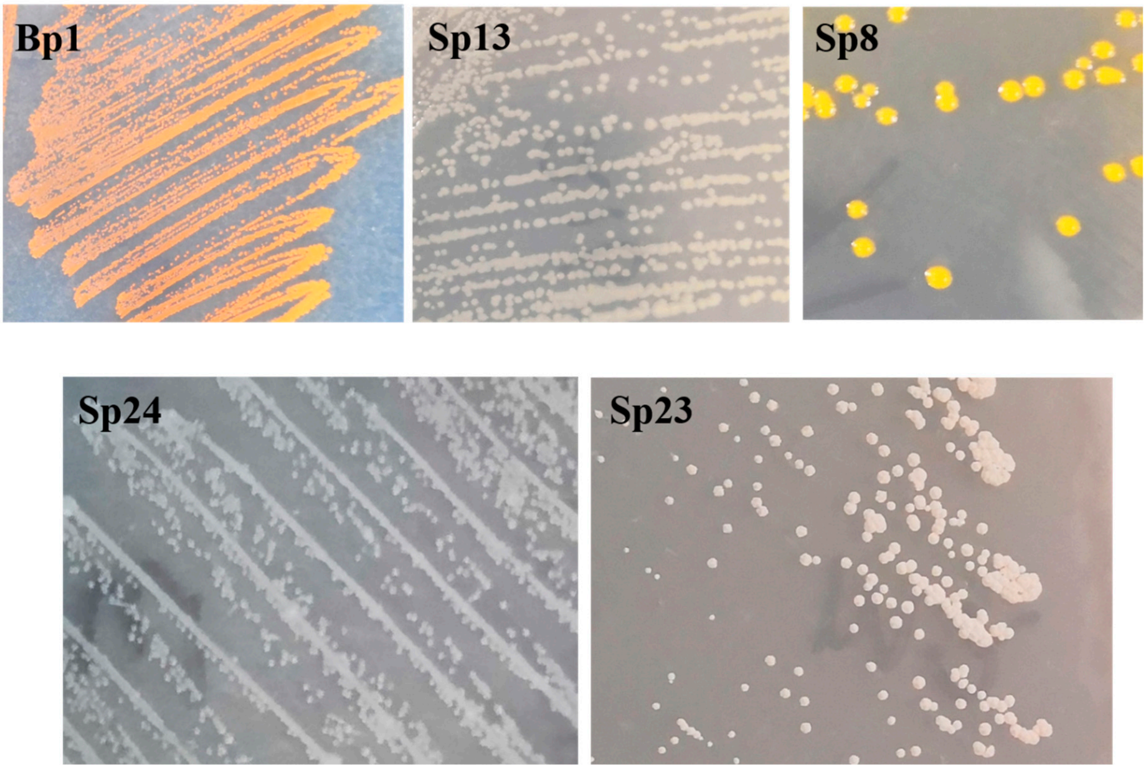

Figure S1. Colonies of the five bacterial strains on M8 agar medium.

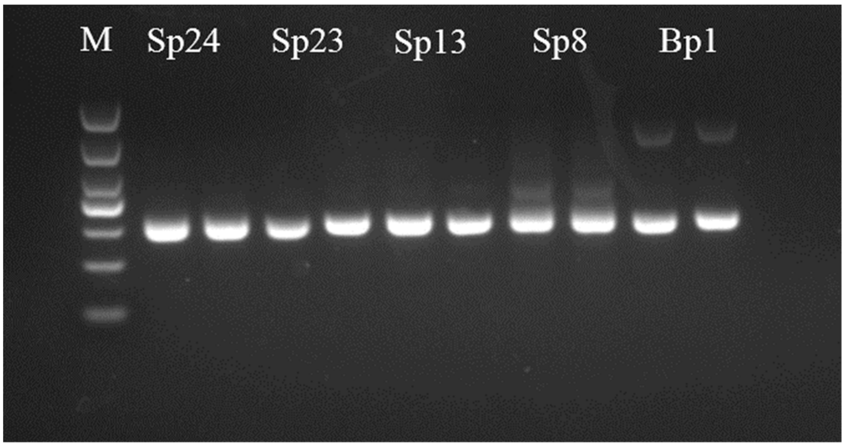

Figure S2. Nested PCR round of RHD genes of the studied isolates. The reaction was run in duplicate for each bacterial strain.

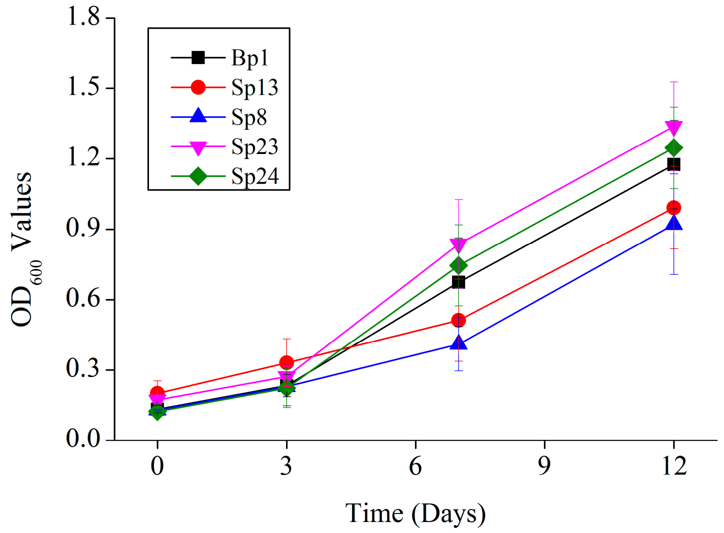

Figure S3. Growth rate of the five bacterial strains during the degradation of pyrene. The OD_{600} value of the negative control group has been subtracted from the OD_{600} values of these groups at the corresponding stage.

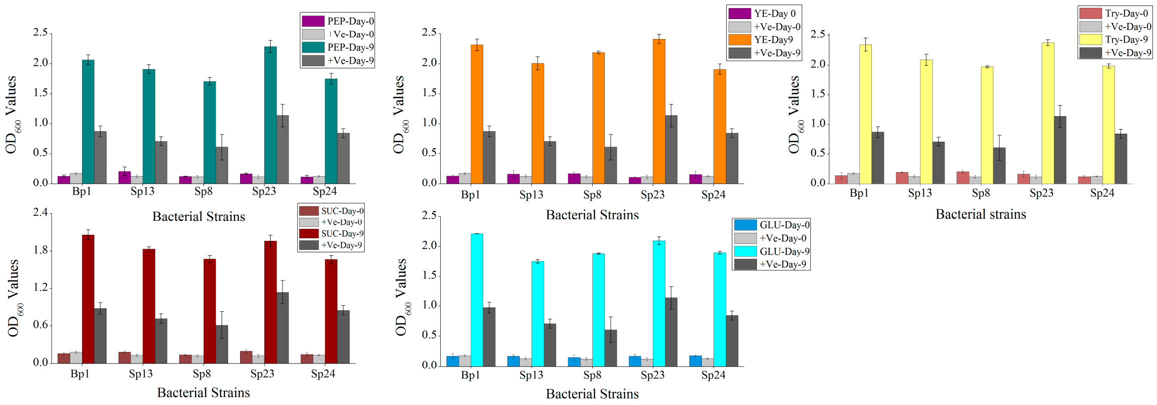

Figure S4. Bacterial growth in terms of OD_{600} values during the degradation of pyrene in the presence of additional carbon and nitrogen sources. The OD_{600} value of the negative control group has been subtracted from the OD_{600} values of these groups at the corresponding stage.

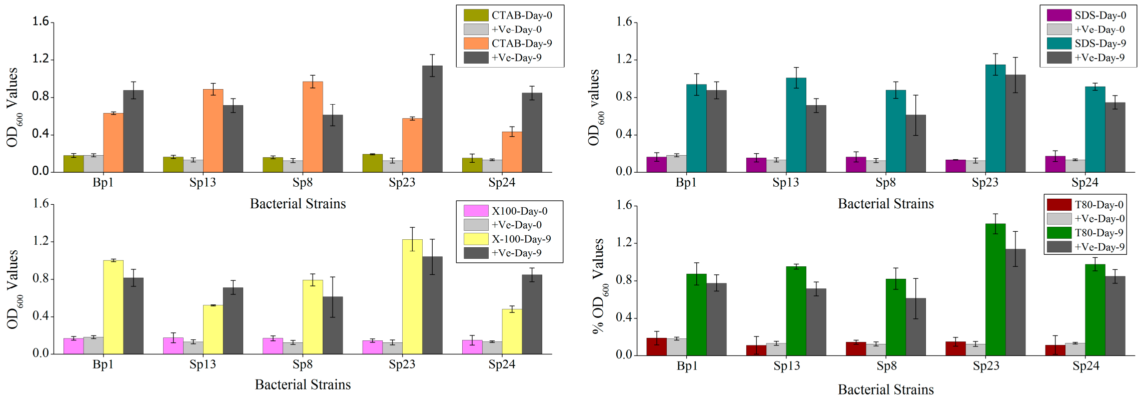

Figure S5. Growth of the five bacterial strains during pyrene degradation in the presence of different kinds of surfactants. The OD_{600} value of the negative control group has been subtracted from the OD_{600} values of these groups at the corresponding stage.

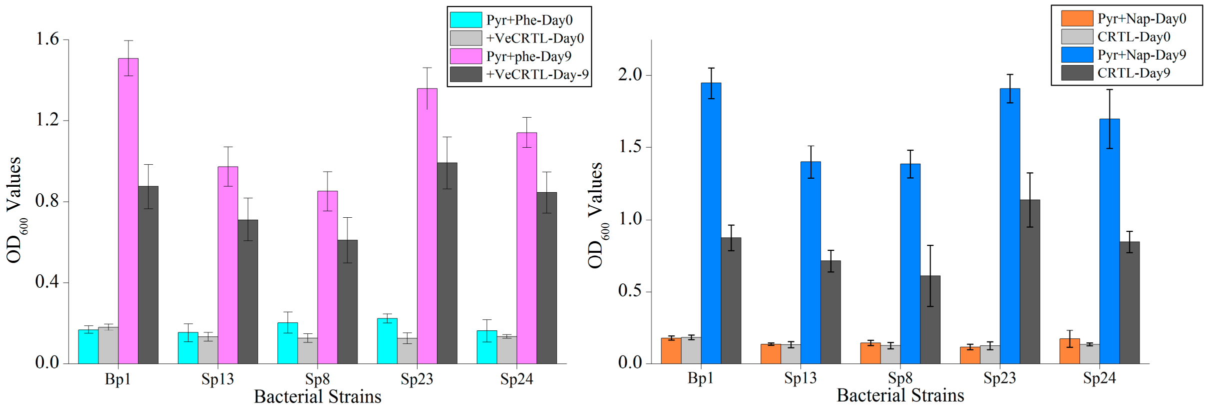

Figure S6. The growth pattern of the five bacterial strains during pyrene degradation amended with phenanthrene and naphthalene. The OD600 value of the negative control group has been subtracted from the OD600 values of these groups at the corresponding stage.

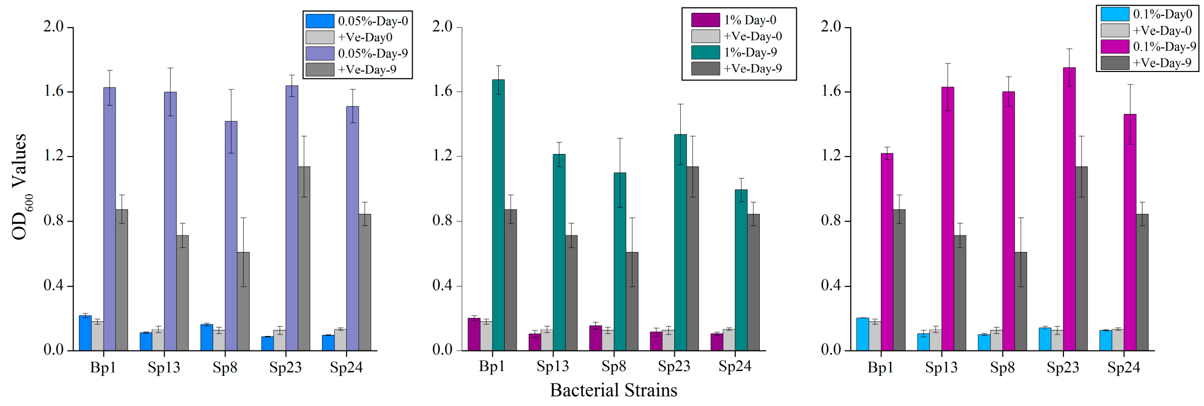

Figure S7. Bacterial growth pattern of the five study isolates during pyrene degradation in the presence of different concentrations of NPK fertilizer. The OD600 value of the negative control group has been subtracted from the OD600 values of these groups at the corresponding stage.

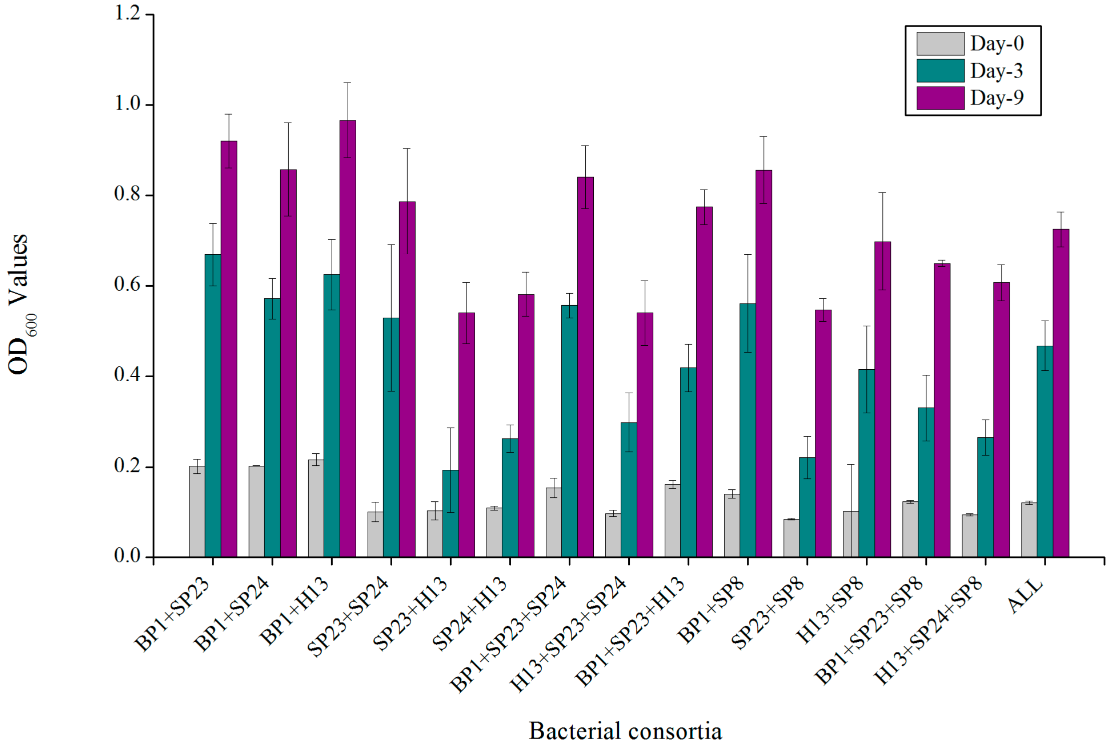

Figure S8. Growth pattern of different consortia during pyrene degradation. The OD_{600} value of the negative control group has been subtracted from the OD_{600} values of these groups at the corresponding stage.
